# Association between patient factors and hospital completeness of a patient-reported outcome measures program in joint arthroplasty, a cohort study

**DOI:** 10.1186/s41687-022-00441-2

**Published:** 2022-04-05

**Authors:** Ian A. Harris, Yi Peng, Kara Cashman, Ilana Ackerman, Emma Heath, Neville Rowden, Stephen E. Graves

**Affiliations:** 1 Australian Orthopaedic Association National Joint Replacement Registry (AOANJRR), Adelaide, Australia; 2 Ingham Institute for Applied Medical Research, South Western Sydney Clinical School, UNSW Sydney, Liverpool Hospital, Liverpool, NSW Australia; 3 South Australian Health and Medical Research Institute (SAHMRI), Adelaide, SA Australia; 4 School of Public Health and Preventive Medicine, Monash University, Melbourne, VIC Australia; 5 St George Private Hospital, South Street, Kogarah, NSW Australia

## Abstract

**Background:**

The collection of patient-reported outcome measures (PROMs) following arthroplasty is common. PROMs data collection programs seek to maximise completeness in order to minimise selection bias and optimise representativeness of the sample attained. We aimed to determine if patient factors influence variation in PROMs program completeness between-hospitals.

**Methods:**

Using data from a national arthroplasty registry PROMs program, we tested for associations between patient characteristics (age, sex, body mass index [BMI] and American Society of Anaesthesiologists [ASA] class) and both potential completeness (registration completeness: the proportion of arthroplasty patients that were registered in the PROMs electronic system) and actual completeness (response completeness: the proportion of arthroplasty patients who provided PROMs data) using linear regression.

**Results:**

When using all elective primary total hip, knee or shoulder arthroplasty procedures (N = 31,801) from 43 hospitals as the denominator, overall registration completeness was 52%, varying from 5 to 87% between hospitals. Overall pre-operative response completeness was 46%, varying from 5 to 82% between hospitals. There were no significant associations between hospital-level registration completeness or response completeness and age, sex, BMI or ASA score.

**Conclusion:**

Completeness rates of a PROMs program in arthroplasty varied widely between hospitals but in the absence of a relationship between measured patient factors and completeness rates, the observed variation likely relates to local site factors such as access to patients, local resources and clinician engagement with the program. Efforts to improve the rates of completeness of arthroplasty PROMs programs at individual hospitals may not improve the representativeness of the sample.

## Plain English summary

Hospitals vary in how completely they include and follow up patients when measuring patient-reported health. It is important to know if the completeness of inclusion and follow up by hospitals is affected by patient characteristics. This study addresses that problem by using data from a national patient outcome survey involving over 30,000 patients from 43 hospitals to look at the correlation between certain patient characteristics (like age, sex, obesity and general health) and the rates of inclusion and follow up for each hospital.

Rates of inclusion (registering patients in the program) and follow up rates varied widely between hospitals (from 5 to 87%). The rates of inclusion and follow up were not correlated with patient age, sex, body mass index or their general health. These findings suggest that the variation between hospitals in the rates of inclusion of joint replacement patients in health surveys are not due to differences in patient characteristics. The differences may be due to local hospital factors, such as resources and clinician engagement.

## Introduction

The collection of patient-reported outcome measures (PROMs) to assess health status in people undergoing arthroplasty surgery provides an important patient perspective on the thresholds for, and outcomes from this resource-intensive procedure. The current state of PROMs collection by registries internationally has been recently summarised, with most registries now collecting PROMs [1]. However, response rates to patient-reported health surveys are rarely above 80% and the representativeness of the sample is often overlooked [2, 3]. Although a threshold of 60% has been suggested for completeness in PROMs collection [4], it is not known if attempts to achieve higher completion rates result in more representative samples (i.e., lower selection bias). Completeness may be divided into registration completeness, defined as the proportion of all patients undergoing surgery who are registered in the PROMs program (and therefore assumed to be invited to participate), and response completeness, defined as the proportion of all patients undergoing surgery who respond to the survey. If patient factors (for example, age and sex) are not associated with completeness, high completeness targets may not be necessary to achieve representativeness, reducing administrative and respondent burden as well as resourcing requirements.

Although previous reports have shown differences in the likelihood of an individual patient responding to a survey based on patient factors (for example, age and sex) [5, 6], the association between patient factors and the relative success (when considered in terms of completeness) in implementing PROMs programs between hospitals has not been reported.

This study aimed to measure the association between hospital-level completeness and available patient-level factors by comparing hospitals involved in a national, registry-based arthroplasty PROMs program.

## Methods

The study analysed a convenience sample of observational, routinely collected data. Between July 2018 and April 2020, the Australian Orthopaedic Association National Joint Replacement Registry (AOANJRR) conducted a PROMs pilot study, collecting pre- and 6 months post-operative PROMs data from patients undergoing hip, knee or shoulder arthroplasty. Patients from 43 hospitals across Australia were included. Different hospital types (high and low volume, metropolitan and regional, private and public) and geographical regions across all six Australian states and one territory (ACT) were represented. The study was nested within the AOANJRR, a national registry that validates more than 97.8% of all arthroplasty procedures performed in Australia [7]. The Australian Orthopaedic Association National Joint Replacement Registry (AOANJRR) was established in 1999 and achieved complete national coverage of all hospitals in 2003. No funding was received for this study.

PROMs data included joint-specific (Oxford Knee Score and Oxford Hip Score) pain and function scores [8], EQ5D-5L quality of life survey [9], joint pain, pre-operative expectations and post-operative satisfaction and perceived change [10]. PROMs data were entered directly using a purpose-built, web-based platform, either by the patient (in clinic or by following links provided by smartphone message or email) or by staff who contacted patients by telephone. Telephone follow-up was only used for patients who did not directly enter data electronically into the web-based platform in clinic or following electronic reminders, or when no means for electronic follow up was recorded. All electronic reminders and telephone calls and all post-operative follow up were administered centrally, within the registry, not at individual hospitals. Individual PROMs data were matched to routine registry data pertaining to the relevant arthroplasty procedure (procedure date, type of procedure, procedure side, age, body mass index [BMI] and American Society of Anaesthesiologists [ASA] physical status classification [11]). Patient characteristics were restricted to the variables available to the registry.

Patient inclusion in the PROMs program involved a two-stage process. The first step was registration in the web-based platform, which could be performed by staff or the patient and consisted of minimal data entry (patient name, date of birth, contact details [email and/or phone numbers], joint, side, surgeon, and hospital) and provision of electronic consent. This was followed by electronic PROMs data entry, which occurred at the time of registration or later, using electronic reminders (email or text message) and telephone follow-up for non-responders. A responder was defined as a patient who answered at least one question.

Due to the two-stage process, sample incompleteness was derived from two sources: patients who underwent arthroplasty but were not registered in the PROMs platform, and patients who underwent surgery and were registered, but did not enter PROMs data. Therefore, sample completeness for each hospital was defined in two different ways:

1. ‘Registration’ completeness (the number of patients registered divided by the total number of procedures performed). This provided a measure of the *potential* completeness of the program at each hospital site and overall.
2. ‘Response’ completeness (the number of responders divided by the total number of procedures performed). This included loss of data due to a lack of patient registration (registration completeness, above) and failure to respond to invitations triggered by the registration process. This provided a measure of the *actual* completeness of the program at each site and overall. Response completeness can be measured for any data collection event (e.g., pre-operative and 6 months post-operative)

This analysis is restricted to elective (non-fracture) primary arthroplasty procedures. The total number of procedures (the denominator) was derived from routine registry data. Representativeness for each hospital was measured with respect to the following patient characteristics: age, gender, BMI and ASA score for each joint (hip, knee and shoulder). ASA score was dichotomised into grades 1–2 and grades 3–5, due to low numbers available for individual analysis for ASA grades 1, 4 and 5.

The completeness rates for each hospital were regressed on summary patient characteristics (mean or percentage) for each hospital. Linear regression was performed for all hospitals, with separate models for each measure of completeness (‘registration’ completeness and ‘response’ completeness), and separate models for each patient characteristic. The significance level was set at 0.05. Given there were two time points for data collection, response completeness was analysed separately for response rates pre-operatively and 6 months post-operatively. These analyses were repeated in multiple linear regression in separate models using all patient factors for each outcome. For each model, the model assumptions were checked by examining standard diagnostic plots. Missing data were not imputed as missingness in the outcome (i.e., completeness) was the dependent variable and the population was restricted to those providing (non-missing) baseline data.

## Results

There was a total of 31,801 hip, knee or shoulder arthroplasty procedures performed at the 43 participating hospitals. For 16,656 (52.4%) of these procedures, patients were registered into the PROMs program (registration completeness) and for 14,506 (45.6%) procedures, patients provided pre-operative PROMs (response completeness). 1185 patients had more than one procedure registered. Post-operative response completeness was 6479 (36.3%); this was restricted to the 17,887 patients who were at least 9 months post-surgery at 30 April 2020. A breakdown of joint procedure type (hip, knee or shoulder) for registration completeness and response completeness is provided in Table 1. Patient characteristics by joint type are summarised in Table 2. Of those who answered at least one question pre-operatively, 96.4% answered all questions. Of those who answered at least one question at post-operatively, 98.9% answered all questions.

**Table 1:** Joint distribution by pre-operative registration and response completeness

Joint	Total	Registration completeness (pre-operative) N (%)	Pre-operative response completeness N (%)	Post-operative response completeness N (%)
Knee	18,215	9770 (53.6)	8576 (47.1)	3849 (37.3)
Hip	11,998	6273 (52.3)	5451 (45.4)	2425 (36.2)
Shoulder	1588	613 (38.6)	479 (30.2)	223 (25.6)
Total	31,801	16,656 (52.4)	14,506 (45.6)	6497 (36.3)

**Table 2:** Summary of patient characteristics by joint

Patient characteristic	Knees	Hips	Shoulders
Mean (SD) Age (years)	68.1 (9.1)	66.7 (12.0)	71.4 (9.2)
Sex (male) (%)	44.4%	45.8%	43.5%
ASA Grade (1 and 2) (%)	58.1%	62.5%	47.5%
Mean (SD) BMI (kg/m2)	32.1 (6.5)	29.5 (6.1)	30.9 (6.2)

The registration completeness and the preop response completeness for each hospital ranged from 4.8 to 86.7% and from 4.5 to 82.1%, respectively. The post-operative response completeness ranged from 3.8 to 69.4% across the hospitals. On average, 1.75 telephone calls were required for each complete response recorded.

Unadjusted regressions of registration completeness on age, sex, BMI and ASA score showed no significant association between hospital registration completeness and any of the patient characteristics. Regressions for response completeness on these variables also showed no significant associations between pre-operative PROMs or post-operative PROMs completeness and any of the patient characteristics. Multiple regression using all patient factors as covariates (i.e., each patient factor adjusted for all others) showed no significant associations for all patient factors and outcomes (Table 3). No violations to model assumptions were identified.

**Table 3:** Multiple regression of registration and response completeness on patient characteristics

Variable	Registration		Pre-op response		Post-op response	
	Beta Coefficient (95% CI)	P	Beta Coefficient (95% CI)	P	Beta Coefficient (95% CI)	P
Mean Age	− 1.18 (− 6.48, 4.12)	0.65	− 1.62 (− 6.68, 3.44)	0.52	− 2.26 (− 6.07, 1.55)	0.24
Mean BMI	− 2.60 (− 9.81, 4.62)	0.47	0.05 (− 6.84, 6.94)	0.99	− 0.83 (− 5.56, 3.91)	0.73
ASA (1 or 2) Percent	− 0.63 (− 1.50, 0.24)	0.15	− 0.38 (− 1.22, 0.46)	0.36	− 0.37 (− 0.97, 0.23)	0.22
Male Percent	− 0.46 (− 2.10, 1.19)	0.58	− 0.55 (− 2.13, 1.02)	0.48	0.06 (− 1.05, 1.16)	0.92

N = 43 for each model

Registration model R^2^ = 0.06, F Value = 0.60, DF = (4,38), p = 0.67

Pre-op response model R^2^ = 0.05, F Value = 0.51, DF = (4,38), p = 0.73

Post-op response model R^2^ = 0.08, F Value = 0.79, DF = (4,38), p = 0.54

## Discussion

This study demonstrates that the between-hospital variation in completeness of a PROMs collection program in arthroplasty patients is not explained by variation in patient characteristics between hospitals. The lack of any association indicates that the variation in completeness between hospitals is not due to the patient factors used in this analysis. Factors such as local resources, clinician engagement and models of care (for example, PROMs patient registration methods) and other patient factors may be responsible for the variation in PROMs completeness between hospitals.

The potential clinical relevance of these findings is that high rates of PROMs completeness, which require significant resources and additional costs [6, 12], may not be necessary to provide valid comparisons with other hospitals. However, very low rates of completeness may still be problematic where events are uncommon (such as detecting specific complications) and where greater precision is required due to random sampling error.

A recent review of large studies [2] (over 1000 patients) reporting PROMs follow-up after arthroplasty showed that the rate of follow-up ranged from 11 to 86% between hospitals, similar to the range seen in this study. Registry-based PROMs programs have been shown to vary in response rate from 31 to 88% [13]. However, the relative representativeness of the included studies was not investigated, and this remains an under-reported aspect of peri-operative data collection. A previous report from the AOANJRR showed that, among patients registered in the PROMs program, representativeness did not change by a large amount with varying patient response rates [10]. A description of the relative response rates between electronic and telephone contact has been previously published showing that responders were more likely to be younger, female and healthier but that these differences were mainly seen pre-operatively rather than post-operatively, and that the addition of telephone follow up did not change the representativeness [14]. Since completing an analysis of the pilot PROMs program, the AOANJRR has now stopped using telephone follow up as the cost does not justify better completeness without significant gains in representativeness.

The strengths of this study are the inclusion of both public and private hospitals, and the range of hospital types, geographical locations and sizes. Another strength is the accurate measurement of the reference population, using validated national registry data. The study also included hospitals with a wide range of registration and response completeness, allowing us to assess the effect of patient characteristics on completeness rates across the full spectrum of likely completeness rates.

We acknowledge there may be other unmeasured patient factors that could impact completeness rates. For example, this study did not have access to data on patient factors such as socio-economic status, English proficiency and health literacy which may vary between hospitals and may be associated with the response rate [15]. Problems encountered in the introduction of this specific PROMs program have been previously reported, including language barriers and varying engagement from local staff [16]. Furthermore, it was not possible to analyse the representativeness of the PROMs responses, whereby patients with poor outcomes may be less likely to respond [17]. Any association between post-surgical outcome and response rate would suggest that post-operative retention of patients recruited pre-operatively may be important, whereas initial (pre-operative) recruitment rates may be less important. This may influence the selection of follow-up timing, as response rates are known to decrease over time [14]. The study findings may not be generalisable to other populations. The study assumes that a census (aiming for collection from all patients) is attempted, and the results may not be applicable to registries that use sampling methods. The study is only relevant for hospital level variation in patient characteristics and therefore more important for between-hospital comparisons.

In summary, our study shows that the (large) variation in completeness rates seen between hospitals was not associated with the patient factors used in this analysis. The costs and increasing patient burden associated with improving hospital-level completeness of PROMs may not result in a more representative sample.

## Data Availability

The AOANJRR is declared by the Commonwealth of Australia as a federal quality assurance activity under section 124X of the Health Insurance Act, 1973. This declaration ensures freedom from subpoena and absolute confidentiality of information held by the Registry. A declaration as a Quality Assurance Activity by the Commonwealth Minister of Health prohibits the disclosure of information, which identifies individual patients or health care providers that is known solely as a result of the declared quality assurance activity. External access to and use of de-identified AOANJRR data is permitted but must be in accordance with AOANJRR policies (Ref No POL.S3.3, S3.4, S3.5) available on the registry website: https://aoanjrr.sahmri.com/policies. Requests for data can be made by contacting the AOANJRR Manager: Cindy Turner, Manager AOANJRR, Telephone: + 618 8128 4284, Email: cturner@aoanjrr.org.au.
